# Perception of interprofessional education and educational needs of students in South Korea: A comparative study

**DOI:** 10.1371/journal.pone.0243378

**Published:** 2020-12-08

**Authors:** So Jung Yune, Kwi Hwa Park, Yul Ha Min, Eunhee Ji

**Affiliations:** 1 Department of Medical Education, Pusan National University College of Medicine, Busan, Republic of Korea; 2 Department of Medical Education, Gachon University College of Medicine, Incheon, Republic of Korea; 3 Kangwon National University College of Nursing, Gangwon-do, Republic of Korea; 4 Gachon University College of Pharmacy, Incheon, Republic of Korea; Waseda University, JAPAN

## Abstract

Due to the recent emphasis on the importance of interprofessional education (IPE) in healthcare fields, interest in IPE introduction is increasing in South Korea. The purpose of this study was to examine the differences in perceptions of medical, nursing, and pharmacy students regarding IPE. Also, the study aimed at identifying the priority rankings of educational needs by analyzing the differences between students’ perceptions of the importance level and the present level for each interprofessional competency. A cross-sectional study was carried out using a survey. A total of 1,500 questionnaires were distributed, of which 1,084 were returned (response rate, 72.3%). The participants were 559 medical, 393 nursing, and 96 pharmacy students. The questionnaire comprised items on the students’ perception of IPE and their interprofessional competency. The questionnaire comprised 12 items on their IPE perception and 9 items on their interprofessional competency. These items were developed by examining the content validity by medical educational specialists and conducting a factor analysis for verification. Data were analyzed using the t-test and ANOVA, and Borich’s formula was used to calculate the rank of educational needs.89.6% did not know the meaning of IPE. The difference in students’ perception of IPE was not significant by grade. Further, the level of IPE perception was higher for female than male students and for students who knew the meaning of IPE than those who did not. The nursing students’ perception of the importance, preference, and effectiveness of IPE was the highest, whereas medical students’ perception was the lowest. All students perceived their present level to be lower than the importance level for each interprofessional competency. Interprofessional communication skills (6.791) were highly necessary for students. These results will serve as baseline information for developing IPE programs in South Korea.

## Introduction

The purpose of an interprofessional healthcare team is to provide patient-centered collaborative treatment using the individual expertise of the team members [1]. The healthcare team should mutually cooperate and communicate to provide optimal expert care. Accordingly, students in various healthcare-related fields should maintain close collaborative relationships; however, these relationships often seem to be conflicting. The maintenance of negative attitudes toward one another, ineffective communication, lack of interpersonal skills, stereotyping and lack of understanding regarding other people’s expertise, and a hierarchical organizational culture have been indicated as the causes of conflict that commonly arise in healthcare and medical professions [2,3].

Interprofessional education (IPE) emerged as one of the methods to alleviate the aforementioned problems and improve patient safety and healthcare quality [4]. IPE is expected to be the key factor affecting education in the healthcare and medical fields in the 21st century [5], and one of the ten recommended items for future education in the fields [6]. IPE refers to creating and fostering a learning environment in which students or professionals from two or more healthcare and medical fields learn together, from each other, and about each other and facilitating reflective interactions among learners [7]. The purpose of IPE is to enable students to learn how to function within an interprofessional team and use their knowledge, skills, and values in future medical settings [1]. It enables students to understand other professions [8] and, ultimately, contributes to teamwork, patient safety, patient satisfaction, the reduction of the occurrence rate of medical errors, and the improvement of medical care quality [9–12]. Accordingly, efforts to help students develop the necessary competence to engage in collaborative practices with healthcare professionals from other specialties after graduation form an important aspect of undergraduate education.

IPE has been developmentally implemented in places such as the United States, Canada, and Europe. In the United States, colleges of nursing, dentistry, and pharmacy formed the Interprofessional Education Collaborative (IPEC) in 2009, and IPEC also became a mandatory requirement of the Liaison Committee on Medical Education Accreditation [13]. In Canada, the Interprofessional Education for Collaborative Patient-Centered Practice Initiative was started in 2003. In the United Kingdom, the Centre for the Advancement of Interprofessional Professional Education was established in 1987. These centers developed academic approaches to IPE implementation [6,7]. In recent years, Asia has also seen an increasing amount of interest and research on IPE [14–17].

While, the research on IPE in medical and nursing education has been reported, studies were mostly conducted on the introduction of the IPE concept [18], the current status of IPE education [19], and perception of IPE by medical personnel or professors [20–22]. Additionally, literature reveals that only 15% of professors reported that they knew about IPE [19]. Further, the ratio of professors with experience in IPE was very low, approximately 4% [19]. Most students, during the preclinical course, are exposed to IPE as a special one-day program as an extra curriculum activity, not as a formal course integrated into the core curricula [19,23,24]. After entering clerkship, students are naturally exposed to the clinical setting, learning about interactions with teams and the role of the team indirectly, as an informal or hidden curriculum [25]. Unfortunately, a majority of students have fewer opportunities to experience a systematically designed formal IPE program.

In addition, the Korean Medical Association stated in its ‘‘Korean doctor’s role” published in 2014 that communication and collaboration are the competencies required of physicians [26]. The Korea Association of Medical Colleges proposed interprofessional collaboration and communication among healthcare professions as the learning outcome for medical students in its “Learning outcomes of basic medical education: Human and society-centered” published in 2017 and recommended that medical schools should teach it to their students [27]. IPE has been attracting attention from medical and nursing schools in South Korea; however, it is fast becoming an important consideration for pharmacy colleges, as well. Recently, South Korean pharmacy colleges reorganized the school system from a four-year to a six-year program and emphasized the importance of developing clinical pharmacology to ensure the production of pharmacists with clinical practice competency [28]. Studies on IPE led by pharmacy colleges are increasing [28], but research remains insufficient in South Korea.

The importance of IPE is gradually increasing in South Korea [19]. Although Korean universities are recommended to adopt IPE, such efforts are in their beginning stages. To introduce and implement these programs jointly held by several health-related colleges, such as medicine, nursing, and pharmacy, it is necessary to explore the differences in their perceptions of IPE. These differences can be challenges in IPE [29,30]. Therefore, it is important to explore their perceptions of IPE to help to design this program. However, little is known about this issue, except for a recent study with nursing students in South Korea [31].

Furthermore, understanding students’ perception of their IPE competency to introduce IPE in universities helps prioritize educational needs because the difference between the importance level of competence (what should be) and present competence level (what is) is a criterion for determining the relative importance of education [32]. If there is a large difference between the level of importance and the present level of competence as perceived by students, the program will be designed prioritizing the development of that competency. Therefore, it is important to explore the level of competence perceived by students. Although a previous study explored faculty members’ perceptions [20], no one has yet investigated students’ perceptions of interprofessional competencies in South Korea.

Exploring students’ perceptions before introducing IPE programs would help design these programs according to the needs of all students. This study takes the first step in this direction by examining their perceptions of IPE and their interprofessional competencies. To this end, we examined the differences in the perceptions of IPE among medical, nursing, and pharmacy students in South Korea. In addition, we analyzed the differences between the importance level and their present level in interprofessional competency as perceived by students to derive the priority of educational needs. Finally, this study intended to serve as baseline information for developing IPE programs.

## Methods

### Study design

A cross-sectional study was conducted using a survey, which was completed by medical, nursing, and pharmacy students in South Korea.

### Sample size

The sample size was calculated by G-power 3.1.9.3 analysis software [33]. The number of participants was higher than the recommended number of 252, calculated by an effect size of 0.25 and a power of 0.95, which is required for the analysis of variance (ANOVA) (three groups). For the three comparison groups, the minimum necessary sample size was 84 for each group, and the recommended sample size was achieved in this study.

### Data collection

Students from five medical, nursing, and pharmacy schools participated in this study. The IPE program had never been implemented as a curriculum in their schools; thus, the respondents had no prior IPE experience. A letter of permission was sent to each school; after obtaining permission, the survey was scheduled. The purpose of the study and details regarding anonymity and confidentiality were explained to the participants. They were asked to read and answer the questionnaire items by themselves. All participants had the right to withdraw at any time. We conducted the survey among students of medicine, nursing, and pharmacy from April 1 to 30, 2018 in Korea. It took students about 20 minutes to complete the survey. A total of 1,500 questionnaires were distributed, of which 1,084 were returned (return rate, 72.3%). Among the returned questionnaires, 36 were excluded since they contained missing or insincere responses and 1,048 were analyzed. This study was approved by the Gil Medical Center Institutional Review Board of Gachon University (IRB approval no., GCIRB-2018-150).

### Instruments

#### Survey of the perception of interprofessional education

To investigate students’ perception of IPE, related literature was reviewed [20,34]. A total of 17 items were devised to explore the perception of IPE after the content validity was examined twice by two medical education specialists with IPE experience. The construct validity of this scale was tested with factor analysis. It was run using the principal components extraction method with Varimax rotation. To determine whether the data were appropriate for factor analysis, the Kaiser-Meyer-Olkin (KMO) measure of sampling adequacy and Bartlett’s test of sphericity were performed on 17 items. The KMO measure of sampling adequacy was 0.945 and Bartlett’s test result was significant (χ2 = 10,994.382, df = 66, p = 0.000), which indicated that variables were independent of each other and the collected data were appropriate for factor analysis. After removing the five items that had cross-loading and factor loading lower than 0.5, a total of 12 items were loaded under three extracted factors. For better results, a practically significant threshold of factor loadings should be greater than 0.5 [35]. About 79.19% of the total variance was explained by these factors. The naming strategy we used was to consider the central concept of all items under each factor. Therefore, factors 1, 2, and 3 were named IPE effectiveness (six items), IPE preference (four items), and IPE importance (two items), respectively. All the Cronbach’s α coefficients of sub-factors were satisfactory (Table 1). Each item was scored using a 5-point Likert scale (with values ranging from 5, strongly agree, to 1, strongly disagree), and higher scores indicated higher levels of perception.

**Table 1:** Factor loadings of IPE perception items for students.

	Factor			Cronbach’s α
	I	II	III	
6. IPE is helpful for students to understand the medical care system	.826	.275	.213	.941
7. IPE is helpful for students to understand the roles and responsibilities of those in other healthcare field within the medical care environment	.811	.310	.223	
4. IPE will improve communication among the students of various fields within the clinical practices.	.801	.303	.298	
8. IPE allows for students to respect and trust students from other field of healthcare	.749	.365	.264	
3. IPE will improve collaborative practice among the students in various field of the clinical environment.	.746	.304	.315	
5. IPE is helpful for students to understand the limitations of their field within the clinical environment.	.738	.310	.272	
10. I prefer lectures (for example, team teaching) that are taught jointly by professors of other healthcare field.	.237	.868	.172	.899
9. I prefer to take lectures with students in other healthcare field	.343	.799	.207	
11. It is advisory to recommend students to participate in subjects related to IPE.	.398	.683	.365	
12. I am willing to participate in programs related to IPE within the field of healthcare.	.437	.680	.308	
2. IPE should be one of the educational goals of our department.	.279	.328	.830	.815
1. It is important for our department to provide students with the opportunities to participate in IPE.	.446	.247	.752	
Variance(%)	37.017	25.621	16.512	
Cumulative(%)	37.017	62.637	79.149	

Factor I: Effectiveness of IPE, Factor II: Preference of IPE, Factor III: Importance of IPE.

#### Survey on the perception of interprofessional education collaborative competency

To determine IPE competency, nine interprofessional competencies proposed by Yune et al. [20] were used. The nine competencies are collaborative leadership, interprofessional communication, interprofessional empathic ability, interprofessional conflict resolution ability, interprofessional decision-making ability, interprofessional problem-solving ability, understanding the role of other professionals in interprofessional collaboration, understanding the role of self in interprofessional collaboration, and legal and ethical judgment ability. Students evaluated the importance level of competence (what should be) and their present competence level (what is) on a 5-point scale for each competency.

### Data analysis

First, data were analyzed to examine general participant characteristics. Second, to examine the differences in perceptions of IPE among medical, pharmacy, and nursing students, t-test and ANOVA were performed. The Scheffé test was performed as a post-hoc test in ANOVA. Third, to clarify the importance level (what should be) and present level (what is) of IPE competency and test differences in the form of averages, paired t-tests were performed. Finally, the order of educational needs was calculated using Borich’s formula (educational needs = Σ(RCL-PCL)×$\bar{RCL}$/N, RCL is the importance level of competency, PCL is the present level of competency, $\bar{RCL}$ is the mean scores for importance level and N is the number of cases) [32].

## Results

### Students’ socio-demographic characteristics

The participants comprised 559 (53.3%) medical, 393 (37.5%) nursing, and 96 (9.2%) pharmacy students. They included 226 (21.6%) first-year, 221 (21.1%) second-year, 302 (28.8%) third-year, and 229 (28.5%) fourth-year students. The numbers of male and female students were 435 (41.5%) and 613 (58.5%) respectively. Finally, 109 participants (10.4%) responded that they “know” the meaning of IPE, whereas 939 participants (89.6%) responded that they “do not know” (Table 2).

**Table 2:** General characteristics of students.

		Medical students	Nursing students	Pharmacy students	Total
Grade	1^st^	113	92	21	226
	2^nd^	107	96	18	221
	3^rd^	171	105	26	302
	4^th^	168	100	31	299
Gender	Male	354	55	26	435
	Female	205	338	70	613
I know about the meaning of IPE	Yes	38	58	13	109
	No	521	335	83	939
Total		559	393	96	1,048

### Students’ perception of IPE

The participants’ perceptions of the importance (F = 2.264, p = 0.079), preference (F = 1.812, p>.05), and effectiveness (F = 1.839, p>.05) of IPE were not significant by grade. In the case of differences by gender, female students’ perception of the importance (t = -4.627, p < .001), preference (t = -4.258, p < .001), and effectiveness (t = -5.568, p < .001) of IPE was significantly higher than male students’ perception.

The students who knew the meaning of IPE was significantly higher for the importance (t = 4.291, p < .001), preference (t = 3.661, p < .001), and effectiveness (t = 4.169, p < .001) of IPE than that of students who did not. Finally, significant differences in the perception of the importance (F = 93.016, p < .001), preference (F = 84.352, p < .001), and effectiveness (F = 121.031, p < .001) of IPE by major were found, and the nursing students had the highest perception for all factors, whereas medical students had the lowest (Table 3).

**Table 3:** Perceptions towards interprofessional education among students.

		N	Importance	F/p, t/p, Scheffé test	Preference	F/p, t/p, Scheffé test	Effectiveness	F/p, t/p, Scheffé test	Total	F/p, t/p, Scheffé test
Grade	1^st^	226	3.82±0.74	F = 2.264 p>.05	3.54±0.83	F = 1.812 p>.05	3.94±0.67	F = 1.839 p>.05	3.78±0.65	F = 1.965 p>.05
	2^nd^	221	3.65±0.87		3.36±0.92		3.83±0.83		3.64±0.80	
	3^rd^	302	3.66±0.80		3.40±0.85		3.79±0.77		3.63±0.73	
	4^th^	299	3.66±0.92		3.38±0.94		3.88±0.82		3.68±0.80	
Gender	Male	435	3.55±0.87	t = -4.627 p < .001	3.28±0.88	t = -4.258 p < .001	3.70±0.79	t = -5.568 p < .001	3.53±0.75	t = -5.457 p < .001
	Female	613	3.79±0.80		3.51±0.87		3.97±0.75		3.79±0.74	
Major	Medicine ^a)^	559	3.40±0.85	F = 93.016 p < .001	3.11±0.88	F = 84.352 p < .001	3.55±0.78	F = 121.031 p < .001	3.38±0.75	F = 127.846 p < .001
	Nursing ^b)^	393	4.10±0.66	b>a, b>c, c>a	3.81±0.75	b>a, c>a	4.26±0.57	b>a, b>c, c>a	4.08±0.56	b>a, b>c, c>a
	Pharmacy ^c)^	96	3.73±0.74		3.61±0.81		3.99±0.69		3.81±0.66	
knowing the meaning of IPE	Yes	109	3.97±0.71	t = 4.291 p < .001	3.71±0.78	t = 3.661 p < .001	4.11±0.65	t = 4.169 p < .001	3.95±0.61	t = 4.745 p < .001
	No	939	3.66±0.85		3.38±0.79		3.82±0.78		3.65±0.76	

### Differences in the perceived importance level and present level of interprofessional competency

There was a significant difference in the perceived importance level for all interprofessional competencies between medical, nursing, and pharmacy students (p < .001). Nursing students perceived the highest level of importance for all competencies (p < .001) (Table 4).

**Table 4:** Differences between perceived importance and perceived present level of interprofessional competency.

	Perceived importance level						Perceived present level						All students			
	Medical students ^a)^	Nursing students ^b)^	Pharmacy students ^c)^	F	p	Scheffé test	Medical students	Nursing students	Pharmacy students	F	p	Scheffé test	Perceived importance	Perceived present level	t	p
Collaborative leadership	3.99±0.85	4.41±0.68	3.75±0.97	42.126	< .001	b>a, b>c, a>c	2.83±0.89	2.77±0.92	2.58±0.85	3.280	< .05	a>c	4.13±0.84	2.78±0.90	37.275	< .001
Communication skills	4.22±0.84	4.73±0.52	4.46±0.70	56.517	< .001	b>a, b>c, c>a	2.86±0.92	2.93±0.94	3.06±1.10	2.181	>.05		4.43±0.76	2.90±0.95	43.550	< .001
Empathic skills	3.97±0.93	4.49±0.66	4.06±0.94	46.051	< .001	b>a, b>c	2.97±0.98	3.07±0.98	2.82±0.96	2.932	>.05		4.17±0.88	2.99±0.98	32.132	< .001
Conflict solving skills	4.14±0.87	4.62±0.60	4.54±0.58	50.945	< .001	b>a, c>a	2.86±0.88	2.79±0.85	3.15±1.10	6.202	< .01	c>a, c>b	4.36±0.79	2.86±0.90	42.561	< .001
Decision making skills	4.04±0.90	4.55±0.65	3.98±0.97	48.168	< .001	b>a, a>c	2.83±0.90	2.75±0.85	2.69±0.87	1.522	>.05		4.23±0.86	2.79±0.88	39.991	< .001
Problem solving skills	4.14±0.87	4.64±0.57	4.40±0.66	50.011	< .001	b>a, b>c, c>a	2.81±0.89	2.73±0.86	3.11±1.07	7.073	< .01v	c>a, c>b	4.35±0.79	2.81±0.90	43.200	< .001
Understanding the roles of other professionals	4.02±0.89	4.49±0.68	3.99±0.95	42.170	< .001	b>a, b>c	2.80±0.92	2.82±0.91	2.74±0.93	0.319	>.05		4.19±0.85	2.80±0.92	37.557	< .001
Understanding their own roles within the collaborative practice	4.13±0.87	4.61±0.60	4.42±0.61	46.013	< .001	b>c, c>a	2.95±0.90	3.05±0.88	3.27±1.04	5.627	< .01	c>a	4.33±0.79	3.01±0.91	38.783	< .001
Legal ethical decision-making skills	4.05±0.95	4.54±0.72	3.97±1.02	40.644	< .001	b>a, b>c	2.86±1.00	2.83±0.95	2.93±0.98	0.371	>.05		4.22±0.91	2.85±0.98	35.547	< .001

The perceived present level of interprofessional competency was significantly different in collaborative leadership (F = 3.280, p < .05), conflict solving skills (F = 6.202, p < .01), problem solving skills (F = 7.073, p < .01), and understanding their roles within the collaborative practice (F = 5.627, p < .01). In general, the present level of competence was perceived by pharmacy students as high, whereas nursing students perceived it as low.

All students perceived their present competence level to be significantly lower than their perceived level of importance for interprofessional competency (p < .001).

### Analysis of the priority of IPE education needs

For all students, the priority of educational needs was determined using Borich’s needs formula for the development of IPE programs. The results showed that the highest priority was assigned to communication skills (6.791), followed by problem-solving skills (6.707), conflict-solving skills (6.517), decision-making skills (6.092), and understanding the roles of other professionals (5.833). The priority in terms of the different majors’ needs was communication and problem-solving skills for medical students, problem-solving and communication skills for nursing students, and conflict-solving and communication skills for pharmacy students (Table 5).

**Table 5:** Educational needs assessment of interprofessional education.

	Interprofessional competency	Medical students		Nursing students		Pharmacy students		All students	
		Needs assessment scores	Priority	Needs assessment scores	Priority	Needs assessment scores	Priority	Needs assessment scores	Priority
1	Collaborative leadership	4.626	8	7.233	7	4.375	8	5.530	8
2	Communication skills	5.773	1	8.505	2	6.223	2	6.791	1
3	Empathic skills	3.966	9	6.377	9	5.036	6	4.920	9
4	Conflict solving skills	5.273	3	8.482	3	6.339	1	6.517	3
5	Decision making skills	4.920	4	8.173	4	5.140	4	6.092	4
6	Problem solving skills	5.507	2	8.841	1	5.632	3	6.707	2
7	Understanding the roles of other professionals	4.892	5	7.512	6	4.987	7	5.833	5
8	Understanding their own roles within the collaborative practice	4.882	6	7.184	8	5.061	5	5.723	7
9	Legal ethical decision-making skills	4.807	7	7.735	5	4.134	9	5.779	6

## Discussion

Although the necessity of the IPE implementation in the healthcare field was emphasized recently, IPE research has not yet been actively conducted in South Korea. Even basic research on students’ IPE perceptions is lacking. This study analyzed the differences in the IPE perceptions of medical, nursing, and pharmacy students, as well as the differences between the importance level (what should be) and their present level (what is) for each interprofessional competency perceived by students.

The majority of the students (88.5%) answered that they did not know the meaning of IPE. The finding confirms that IPE has not yet been effectively implemented in South Korea. IPE research on students was first conducted in nursing education, and research is at the stage of investigating students’ IPE attitudes and readiness [31]. In South Korean medical schools, lecture-based IPE is provided to medical students to enable them to understand the characteristics of and work done in other healthcare professionals, including nursing [19], and active IPE programs are rarely implemented [24]. Furthermore, in the pharmacy colleges of South Korea, although the relative importance of clinical pharmacology is gradually increasing, the level of perception of pharmacists as a part of medical staff is low [36]. Hence, their interest in IPE is more insufficient. Meanwhile, a study on professors of medical, nursing, and pharmacy schools demonstrated that 85.2% of them did not know about IPE [20], which was similar to the proportion of students in this study. Such a low perception of IPE among the South Korean academia appears to have affected students’ perception, as well.

The difference in the perceptions of IPE by grade was not significant. Diverse research findings have been reported on this topic, including indications that the perception and attitude toward IPE tend to decline with an increase in grade level [37–40], tend to positively increase with an increase in grade level [41], and display no difference in lower or entire grades [42]. Nonetheless, students’ perceptions of interprofessional learning is generally favorable before their exposure to IPE [43]. However, their interest and attitudes tend to change as the amount of learning increases with an increase in grade level. Consequently, more longitudinal studies are required to determine changes according to grade.

Female students perceived the importance, preference, and effectiveness of IPE more positively than the male students. This finding is consistent with the results of many other studies [43–47]. Compared to male students, female students have a stronger professional identity [43], attribute more seriousness to patient-centered treatment and a collaborative attitude, and tend to have a stronger willingness to accept them [48]. These principles appear to have a positive influence on their attitudes toward IPE. In this study, approximately 55% of the female students were nursing students. More nursing students showed positive attitudes toward IPE compared to other healthcare professionals [37,43], and the high number of nursing students in this study may have influenced the results.

Among all students, nursing students’ perceptions of the importance, preference, and effectiveness of IPE were the highest, whereas medical students’ perceptions were the lowest. The importance of IPE is further emphasized for medical and nursing students because they spend the majority of their time collaborating with others to deliver patient care. However, medical students are more skeptical about IPE than students in other healthcare fields [49] and tend to consider it less important [50]. Medical students consider IPE as a waste of time due to their tight curriculum and excessive academic burden and tend to show less interest in and passion toward IPE implementation. Traditionally, the healthcare system is built on a strict order of rank among healthcare professionals, and Asian countries, including South Korea, have a stronger social hierarchical culture than Western countries [17]. Such medical environments might have influenced the low perception of medical students. On the other hand, like nursing students, pharmacy students’ perception of the importance, preference, and effectiveness of IPE was higher than that of medical students. This result supports several previous findings in this area [51,52]. In the health education environment of South Korea, both pharmacy and medical students still have limited learning opportunities about each other's roles and responsibilities. It has not been long since clinical practice was introduced in hospitals under the 6-year pharmacy curriculum for clinical pharmacology. However, in recent years, from the perspective of patient safety, the importance of collaboration between doctors and pharmacists has been emphasized [53], and it seems that pharmacy students are beginning to understand the importance of IPE. These results will provide evidence to promote the introduction of IPE in pharmaceutical education.

Students perceived their present level to be lower than their perceived importance level for nine interprofessional competencies. Although the importance level of each competency was four points or higher on average, the present level was three points or less on average. This disparity suggests the importance of appropriate education because students considered each competency to be important but perceived their present level to be relatively low. Among all students, nursing students evaluated the highest importance levels for all competencies. Finally, in the present study, nursing students maintained the most positive attitude toward IPE, and this attitude appears to have positively influenced their perception of the importance levels.

Each student had different priorities concerning their educational needs. While medical and nursing students prioritized communication and problem-solving skills, pharmacy students prioritized conflict-solving and communication skills. Students are prepared in these competencies of problem-solving skills, conflict-solving, and communication skills, for collaborating effectively with their future healthcare teams [6]. Particularly in the context of conflict arising among healthcare professionals, these competencies are essential skills to resolve [54]. Although there are differences in educational needs among students, it is thought that these competencies are considered key competencies for collaboration among healthcare professionals. On the other hand, communication skills were commonly considered a priority. Also, for all students, the results of the Borich coefficient calculation revealed that the highest educational need was for interprofessional communication skills. Students perceived the importance level of this competency to be very high, with an average score of 4.44. However, their present level was perceived to be low, with an average score of 2.91, which revealed a significant difference between the two scores. In other words, interprofessional communication skills should be assigned the highest priority in students’ IPE programs. This finding is the same as the finding of a study on medical, nursing, and pharmacy professors [19]. Further, interprofessional communication has been suggested as an important and common competency domain in a comparative study involving many countries [55,56]. Accordingly, systematic programs should be developed to improve students’ present competence levels.

This study is significant as it emphasizes the importance and necessity of IPE implementation in South Korea, promotes interest in IPE research, and provides basic data to develop IPE programs in the future. However, since this study was based on a self-report questionnaire, future studies using qualitative methods, such as interviews, are necessary to conduct an in-depth analysis of students’ perceptions. The data were collected from four medical schools, one nursing school, and two pharmacy schools. Hence, the limitation is that this sample might not be representative of the entire country. As this study is not intended for scale development, it was insufficient to examine the value of CVR (Content Validity Ratio) and CVI (Content Validity Index) for content validity. The development of a valid and reliable standardized tool for the South Korea version that can measure the perception and attitude toward IPE is also required. Further, it is necessary to conduct a follow-up study to develop an IPE program to improve the interprofessional competency required for students and verify its effect.

## Conclusions

The nursing students’ perception of the importance, preference, and effectiveness of IPE was the highest, whereas medical students’ perception was the lowest. Students perceived that their present level of competence was lower than the level of importance for the nine interprofessional competencies. Further, for all students, the highest educational need was for interprofessional communication skills. This result will serve as baseline information for developing future IPE programs
